# *In vitro* evaluation of novel consensus-sequence-generated Brevinin-2 antimicrobial peptides

**DOI:** 10.1128/aem.01553-26

**Published:** 2026-09-14

**Authors:** Colin M. McDowell, Jessica Carder, James Brozik, Jennifer Horton, Douglas R. Call

**Affiliations:** 1 Paul G. Allen School for Global Health, College of Veterinary Medicine, Washington State University 663014, Pullman, Washington, USA; 2 Department of Chemistry, Washington State University 735800, Pullman, Washington, USA; Michigan State University, East Lansing, Michigan, USA

**Keywords:** Brevinin-2, antimicrobial peptide, antimicrobial resistance

## Abstract

Antibiotic-resistant bacteria are a global public health threat that is becoming increasingly difficult to address with conventional therapeutics. Consequently, there is much interest in studying alternative biologics that circumvent antibiotic resistance. The Brevinin-2 family of antimicrobial peptides (AMPs) is a group of naturally occurring molecules that have the potential for high activity and low toxicity. Herein, we investigated the potential of a consensus-sequence-driven approach to Brevinin-2 peptide synthesis and evaluated their action against a panel of multidrug-resistant (MDR) bacteria, including carbapenemase-resistant *Escherichia coli*. We used the positional frequency of amino acids to generate novel synthetic peptides representative of the Brevinin-2 family. Four templates—G30, G33, S33, and G37—were synthesized by standard fluorenylmethyloxycarbonyl (FMOC) chemistry, purified by reverse-phase fast protein liquid chromatography (RP-FPLC), and tested for antibacterial activity and hemolytic toxicity. The results demonstrated a broadly applicable chemical peptide synthesis pipeline for the Brevinin-2 family with a high degree of purity (>90%). Two peptides—S33 and G33—exhibited activity consistent with potential selectivity toward gram-positive and -negative bacteria, respectively, while G37 displays broad-spectrum activity, with growth of a Class B carbapenemase-resistant *E. coli* inhibited at 16 µM and a Class A carbapenemase-resistant *K. pneumoniae* inhibited at 64 µM. G37 acts rapidly, slowing growth within 30 min and fully killing targeted bacteria within 150 min. Although moderate levels of hemolytic toxicity pose a challenge for future development, the consensus-sequence approach toward novel Brevinin-2 AMP discovery via alignment combined with *in vitro* antimicrobial analysis shows promise to test and initially validate other peptides.

**IMPORTANCE:**

The escalating threat of antimicrobial resistance (AMR) demands innovative therapeutic strategies beyond traditional antibiotics. This study demonstrates a systematic, consensus-sequence-driven approach to designing antimicrobial peptides (AMPs) from the naturally occurring Brevinin-2 family, offering a replicable framework for accelerated drug discovery. Our novel peptide G37 exhibits bactericidal activity against carbapenemase-resistant *Escherichia coli* within 150 min while maintaining low hemolytic activity at therapeutic concentrations. The potential preferential activity shown by peptides S33 and G33 for gram-positive versus gram-negative bacteria provides prospective valuable insights into structure-activity relationships that can guide further peptide optimization. By combining computational sequence analysis, standardized solid-phase peptide synthesis, and comprehensive *in vitro* validation, this work establishes a streamlined pipeline for Brevinin-2 peptide development. This methodology addresses the urgent need for alternative antimicrobials while providing a scalable approach to combat multidrug-resistant (MDR) pathogens.

## INTRODUCTION

The increasing prevalence and severity of antimicrobial resistance (AMR) is a rising public health concern (1–3). Although novel antibiotics continue to enter the developmental pipeline, the race between antibiotic discovery and the distribution of antimicrobial-resistant bacteria is not encouraging (4, 5). Consequently, there is a continuing need for novel biologics capable of countering or circumventing classic antibiotic resistance mechanisms.

Antimicrobial peptides (AMPs) are short, amphipathic sequences of amino acids and are an important component of the innate immune system across a wide diversity of taxa (6). Distinct from bacteriocins produced by microorganisms, these host defense AMPs are produced by multicellular organisms as part of their innate immunity. These AMPs commonly display broad-spectrum antimicrobial activity, including against antimicrobial-resistant strains (7). AMPs appear to act in a concentration-dependent manner to disrupt the bacterial membrane through pore formation (8). Unfortunately, this mechanism may lack sufficient specificity at therapeutic concentrations, so they can also be toxic to the host, and they can exhibit chemical instability, which further limits their application in clinical cases (9).

Originally isolated from the Anuran order of frogs and toads, the Brevinin-2 family of AMPs exhibits inhibitory activity against gram-negative and -positive bacteria—including biofilm-forming species such as *Pseudomonas aeruginosa*—while exhibiting relatively low host toxicity (10, 11). These peptides are primarily identified by the presence of a six-residue C-terminal disulfide domain known as a “Rana-box” (10). With more than 100 described members of this family, the Brevinin-2 family serves as a rich sequence space to explore for potential new antimicrobials (12–17). This sequence repertoire allows for robust consensus sequence generation and favorable activity-to-toxicity ratio consideration relative to smaller data sets. In this study, we sought to determine the efficacy of an *in vitro* consensus-sequence-driven approach toward novel Brevinin-2-like AMPs as a eukaryotic host defense mechanism. We identified four novel peptide consensus template sequences (G30, S33, G33, and G37) from multiple sequence alignments of Brevinin-2 family AMPs and synthesized them using standard fluorenylmethyloxycarbonyl (FMOC) solid-phase peptide synthesis (FMOC SPPS), followed by RP-FPLC. This approach has been previously used for other peptides to improve initial peptide traits (18, 19). We selected Brevinin-2GUb as the representative control due to its well-characterized activity profile—exhibited through established MIC data benchmarks—and to provide a reference for comparison (20, 21). To evaluate the efficacy of this approach, we treated a panel of six gram-negative and -positive bacteria with different antibiotic resistance profiles with G30, S33, G33, and G37. We also assessed the toxicity of the generated peptides by quantifying lysis of sheep red blood cells at various concentrations of treatment. Lastly, we evaluated the killing kinetics necessary to achieve a bactericidal dose at different time points for each peptide. Ultimately, this project sought to provide insights into a Brevinin-2 developmental pipeline through a combined *in silico* design, synthetic peptide production, and *in vitro* microbiological and toxicity screening so that we might have a richer understanding of AMP design within the Brevinin-2 family.

## MATERIALS AND METHODS

### Peptide design and sequence generation

Peptide sequences templates were generated using a consensus-sequence modification strategy accounting for variation within the Brevinin-2 family (19, 22). All peptides were designed to exhibit α-helical secondary structure based on helical wheel projection and I-TASSER computational structural models (Fig. S5 and S6; Table 1; 23–26). In brief, 80 Brevinin-2 sequences, obtained from existing AMP databases (12–17), were clustered into four groups according to sequence length and reported activity (Fig. S1 to S4). Each Brevinin-2 group was aligned according to the positional frequency of each amino acid in a multiple sequence alignment using ClustalX2 software to generate a consensus sequence (Fig. S1 to S4; Tables S1 to S4) (22). Peptide G30 was designed from eleven, thirty-residue sequences with nine conserved residues. Peptide G37 was designed from nine, thirty-seven residue sequences with eleven conserved residues. Peptide S33 was designed from twenty, thirty-three residue sequences with seven conserved residues and a universal serine at position one. Peptide G33 was designed from forty, thirty-three residue sequences with three conserved residues and a universal glycine at position 1. The sequence of Brevinin-2GUb was used as a positive control for all groups (20, 21).

**TABLE 1:** Summary of novel peptides and their physicochemical properties^*a*^

Peptide	Sequence	Length	Net charge	Hydrophobicity (%)	µH	MW (Da)
G30	GAFGDTLKGVAKTAALKLLNKAQCKLSGTC	30	+4	47	0.076	3008.6
S33	SFLGTVKDLAIGAAKSAGQSVLKTLSCKLSKTC	33	+4	45	0.122	3327.0
G33	GLLDTFKNVAKNAAKSAGVSLLNTLSCKLSKTC	33	+4	45	0.121	3397.0
G37	GLMSVFKGVLKTAGKHIFKNVGGSLLDQAKCKISGQC	37	+5	43	0.149	3864.6

^*a*^ Summary of novel peptide sequences and some expected physicochemical characteristics. Molecular weight (MW) is expressed as g/mol. The mean hydrophobic moment (µH) is a measure of a peptide’s amphiphilicity and estimated by Heliquest (23). The hydrophobicity of the peptide is the percentage of hydrophobic residues present in the sequence. Peptides S33 and G33 were further distinguished by the universal N-terminal residue (Ser vs Gly) in addition to the other considerations.

### Peptide synthesis

The peptides were synthesized by standard 9-fluorenylmethoxycarbonyl (FMOC) solid-phase peptide synthesis (SPPS) using an APEX 396 peptide synthesizer (AAPPTEC). First, each amino acid used in the synthesis was solubilized in dimethylformamide (DMF) to prepare 0.4 M solutions. Next, the FMOC-Cys(Trt)-Wang resin (binding capacity = 0.572 mmol/g) was added to reaction wells. Proceeding from the C-terminus to the N-terminus, the resin was sequentially swelled in 1.5 mL DMF, deprotected with 1.5 mL 20% piperidine in DMF, washed with 1.5 mL DMF, coupled with 1.5 mL of the next subsequent amino acid through 1.0 M HBTU (O-(benzotriazol-1-yl)-N,N,N′,N′-tetramethyluronium hexafluorophosphate) and 1.0 M DIPEA (N,N-diisopropylethylamine) catalysis, and washed clean with 1.5 mL DMF. Upon completion of the peptide chain, the resin and sidechain protecting groups were cleaved for 2 h while shaking at 200 rpm with 4.0 mL of freshly prepared Reagent K cleavage solution (82.5% trifluoroacetic acid [TFA], 5% phenol, 5% water, 5% triisopropyl silane [TIPS], and 2.5% 1,2-ethanedithiol [EDT]) through vacuum filtration into a −20°C diethyl ether solution. Peptides were washed by cold diethyl ether three times using centrifugal separation (500 × *g* for 1 min), dried in a centrifugal CentriVap (Labconco), and stored at −20°C.

### Peptide purification

Peptides were dissolved in buffer A (99.9% autoclaved Milli-Q H_2_O and 0.1% TFA) and brought to a stock concentration of 1.0 mg/mL by using an analytical balance. The peptides were purified by RP-FPLC chromatography by using an ÄKTA avant chromatography system (GE). First, 10 μL of the solution was injected onto a Kinetex 250 × 4.6 mm C18 RP analytical column (Phenomenex) and eluted by gradient separation using buffer A and buffer B (99.9% acetonitrile; 0.1% TFA) over 15 column volumes (CV) total. The flow rate was set at 0.5 mL/min, while the detection wavelengths were 214, 254, and 280 nm. Following analytical identification of the polar identity of the peaks of interest, 1 mL of the peptide solution was injected onto a 3-mL Resource RPC column (Cytiva) for preparative purification. Elution started at 20% buffer B and, over 10 CV, it advanced linearly to a percentage buffer B 5% above the peak of interest identified in the analytical chromatogram before continuing to 100% buffer B over 5 CV. The flow rate was set at 1.0 mL/min, and the detection wavelengths were placed at 214, 254, and 280 nm. The peptides were centrifugally dried in a CentriVap and stored at −20°C. Peptide purity was >90%, as determined by the area of the peptide peak relative to the total peak area at 214 nm (Fig. S7).

### Antimicrobial assay

Peptide antimicrobial activity was evaluated by using the broth microdilution assay adapted from methods recommended by the Clinical and Laboratory Standards Institute (CLSI) (27). Peptides were tested against both antibiotic-resistant and -susceptible *Escherichia coli*, *Staphylococcus aureus*, and *Klebsiella pneumoniae* (Table 2). Bacteria were streaked for single colonies on cation-adjusted Mueller-Hinton (MH) agar plates and grown overnight at 37°C. Colonies were picked from the plates to prepare a 0.5 McFarland solution in 0.5% saline solution. The McFarland solutions were diluted 1:20 in MH media in preparation for the assay, providing a final concentration of ~5 × 10^5^ CFU/mL.

**TABLE 2:** Summary of bacterial strains and their resistance profiles^*a*^

Organism	Strain	Resistance phenotype	Resistance mechanism	Ambler class	Reference
*E. coli*	ATCC 25922	Susceptible	None		(27)
*E. coli*	NCTC 13476	Carbapenem-resistant	IMP-type metallo-β-lactamase	B	(28)
*K. pneumoniae*	ATCC 700603	ESBL-producing	SHV-18 + OmpK35/OmpK37 porin loss	A (ESBL)	(29)
*K. pneumoniae*	NCTC 13438	Carbapenem-resistant	KPC-3 carbapenemase	A	(30)
*S. aureus*	ATCC 25923	Susceptible	None		(27)
*S. aureus*	ATCC 33592	MRSA; gentamicin-resistant	*mecA* (*PBP2a*)		(31)

^*a*^ Summary of evaluated bacterial strains and their characterized resistance profiles. ATCC 25922 and ATCC 25923 serve as fully susceptible controls for gram-negative and -positive bacteria, respectively.

In 96-well round-bottom plates, 0.1 mL of cation-adjusted MH media was added to all wells except the top row. Peptides were solubilized in water containing 0.1% TFA. After adding the peptides to an appropriate volume of cation-adjusted MH media to reach a concentration of 256 µM, 0.2 mL of the solution was added to the top row of the plate. A 1:2 dilution series was performed down the rows of the plate with thorough mixing until row G, at which point 0.1 mL of the row was discarded. Next, 10 µL of the previously prepared dilute bacterial suspension was added to appropriate wells of a 96-well plate (Fig. S8A). The plate was incubated at 37°C for 16–18 h, and the MIC was visually determined as the lowest concentration of the peptide without visible turbidity from bacterial growth (Fig. S8B).

All antimicrobial assays were conducted with a vehicle control (peptide solvent), a blank control (media without bacteria), positive controls (128 µg/mL tetracycline HCl, 128 µg/mL streptomycin sulfate, and 128 µg/mL ampicillin HCl), and a peptide treatment. At least three independent assays were completed, and each assay included three technical replicates.

### Hemolysis assay

The hemolysis assay was adapted from Sæbø et al. (32). In brief, defibrinated sheep blood (25 mL; <2 weeks old; Hardy Diagnostics) was centrifuged at 500 × *g* for 5 min at 4°C and resuspended in 5 mL phosphate buffer solution (PBS; pH = 7) for a total of three times. The final suspension was enumerated with a hemocytometer and diluted to 1 × 10^9^ RBC/mL in PBS. A volume of 100 µL was added to each well of a 96-well polypropylene plate with conical bottoms. The plate was centrifuged at 500 × *g* for 5 min before the supernatant was aspirated. A volume of 100 µL of a treatment solution was added to the appropriate RBC pellet. The plate was incubated at 37°C for 1 h and centrifuged at 500 × *g* for 5 min. The supernatant was transferred into a flat 96-well plate, and the OD_405_ was determined by using a Tecan Spark plate reader. PBS was used as a negative control, and cells with 0.1% (wt/vol) Triton X-100 were used as a positive control. Brevinin-2GUb was included as a reference peptide in each assay for comparison with the novel peptides. At least three independent assays were completed, and each assay included three technical replicates. The LD₅₀ was defined as the dose needed to induce lysis in 50% of the red blood cell population, calculated by nonlinear logistic regression in GraphPad PRISM. For peptides that did not reach 50% lysis at the highest concentration tested (128 µM), the LD₅₀ exceeded the assay range and is reported as >128 µM. Error bars display the calculated standard error of the mean from the replicates. The percent hemolysis was standardized using the equation:


$$\%\;Hemolysis=\frac{OD_{Treatment}-PBS_{Blank}}{OD_{Triton\;X\text{-}100}-PBS_{Blank}}\times 100$$

### Time-killing assay

The previously mentioned bacteria were grown on MH plates at 37°C overnight to isolate single colonies. Colonies were picked from the plates to prepare 0.5 McFarland standard turbidity in 0.5% saline solution, equivalent to 1.5 × 10^8^ CFU/mL. The McFarland solutions were further diluted 1:20 in MH media for an approximate cell density of 7.5 × 10^6^ CFU/mL. In 96-well round-bottom plates, 0.1 mL of MH media was added to all rows except the top row, and 0.2 mL of a 256 µM peptide solution was added to the top row of the plate. A 1:2 dilution series was performed down the rows of the plate with thorough mixing until row G, at which point 0.1 mL of the row was discarded. The final peptide concentration evaluated was 8 µM. Next, 10 µL of the previously prepared dilute bacterial suspension was added to appropriate wells of the 96-well plate. The plate was incubated at 37°C for 30 min before 5-µL aliquots of the bacterial-peptide mixture were plated in triplicate on MH agar plates (Fig. S9A). This process was repeated to obtain relevant time points, with two samples or controls per plate. Plates were incubated overnight at 37°C, and colonies were counted in the morning. If colonies were too numerous to count (≥100 colonies/5 µL), then the aliquot was labeled “too numerous to count” (TNC) or lawn (Fig. S9B). If a given time point was visually determined to have an average of less than one colony at a specific peptide concentration, that time point was specified as the lethal dose-_99_, or time-to-kill. All time-killing assays were performed with a vehicle control (peptide solvent), a blank control (media without bacteria), a positive control (Brevinin-2GUb), and a negative control (media with bacteria alone). At least three independent assays were completed, and each assay included three technical replicates.

### Statistical analysis

All statistical analyses were performed using Microsoft Excel and GraphPad PRISM v. 10.4.1. For applicable biological experiments, we used three technical and three biological replicates. Descriptive measures were used to characterize the semi-quantitative endpoints and did not warrant further inferential statistical testing. Two distinct lethal-dose metrics were used, reflecting the nature of each assay: hemolytic toxicity was quantified as the LD₅₀ (the dose producing 50% lysis), obtained by nonlinear regression of the continuous dose-response curve, whereas bactericidal kinetics were quantified as the LD₉₉—the earliest time point at which viable counts fell below one colony (<1 colony averaged) at a given concentration—a censored threshold read directly from colony enumeration rather than a fitted midpoint. Accordingly, results are presented as means with standard error of the mean (SEM) from three biological replicates, each comprising three technical replicates. Peptide purity was determined by using Cytiva Unicorn 3.4.1 software to divide the peptide peak relative to the total peak area at 214 nm.

## RESULTS

### Peptide design and generation

Four novel Brevinin-2 peptides—G30, S33, G33, and G37—were devised from the conserved sequences generated by the positional frequency of amino acids from four conserved groups of Brevinin-2 peptides. According to previous studies, the key physicochemical properties for successful peptide design within the Brevinin-2 family are net positive charge, hydrophobicity, amphipathicity, and motif maintenance (10, 33). Therefore, all novel sequences were evaluated *in silico* for desirable physicochemical properties and notable Brevinin-2 characteristics (Table 1; Fig. S5 and S6). All peptides maintained the Rana-box motif within the last six residues of the C-terminus regardless of total peptide length and exhibited a net positive charge, moderate hydrophobic identity, and low amphiphilicity. Novel peptides and peptide controls were synthesized by solid-phase peptide synthesis and purified by reverse-phase fast protein liquid chromatography. All peptides were cleaned to >90% purity (Fig. S7).

### Antimicrobial activity

**Fig 1:**
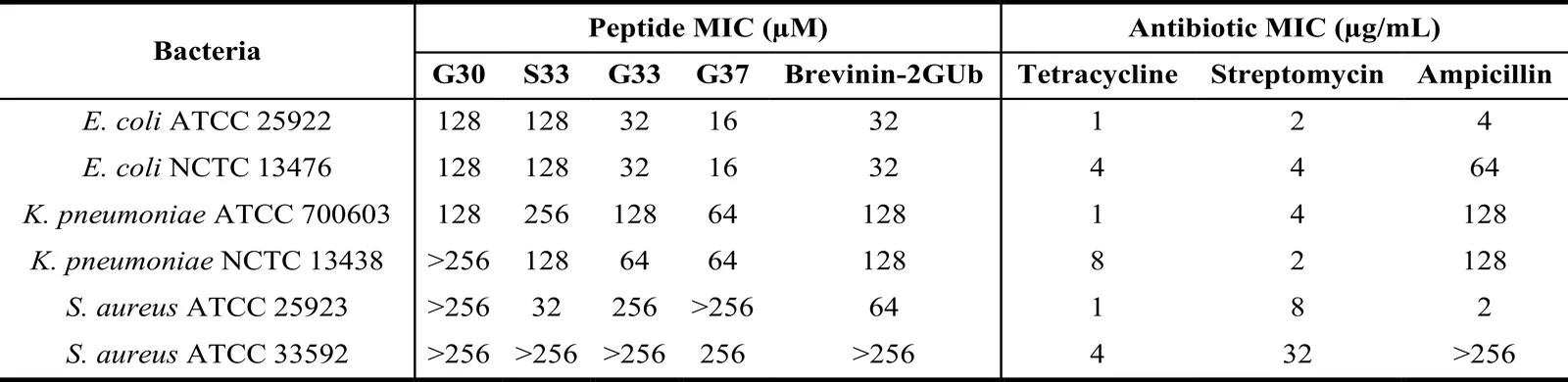
Minimum inhibitory concentrations (µM) of evaluated peptides against gram-positive and -negative microorganisms. Brevinin-2GUb and three standard antibiotics serve as positive controls and references. All experiments were performed in triplicate with three technical replicates.

The MICs of each peptide and reference were determined against reference strains of antibiotic-susceptible and -resistant *E. coli*, *K. pneumoniae*, and *S. aureus* (Fig. 1; Table 2). Peptide G30 displayed limited antimicrobial activity against the evaluated bacteria (128 to >256 µM), whereas G37 presented potent antimicrobial activity (16 to >256 µM). Furthermore, while S33 was consistent with potential differential activity against gram-positive bacteria (32 to >256 µM), G33 was consistent with potential differential activity against gram-negative bacteria (32 to >256 µM). The MIC findings for the Brevinin-2GUb reference matched those of previous work (32 to >256 µM) (20). The MICs for tetracycline, streptomycin, and ampicillin were consistent with published CLSI breakpoints (≤4, ≤8, and ≤8 µg/mL, respectively), while resistant strains displayed elevated MICs consistent with their characterized resistance profiles (Table 2) (27).

### Hemolytic activity

**Fig 2:**
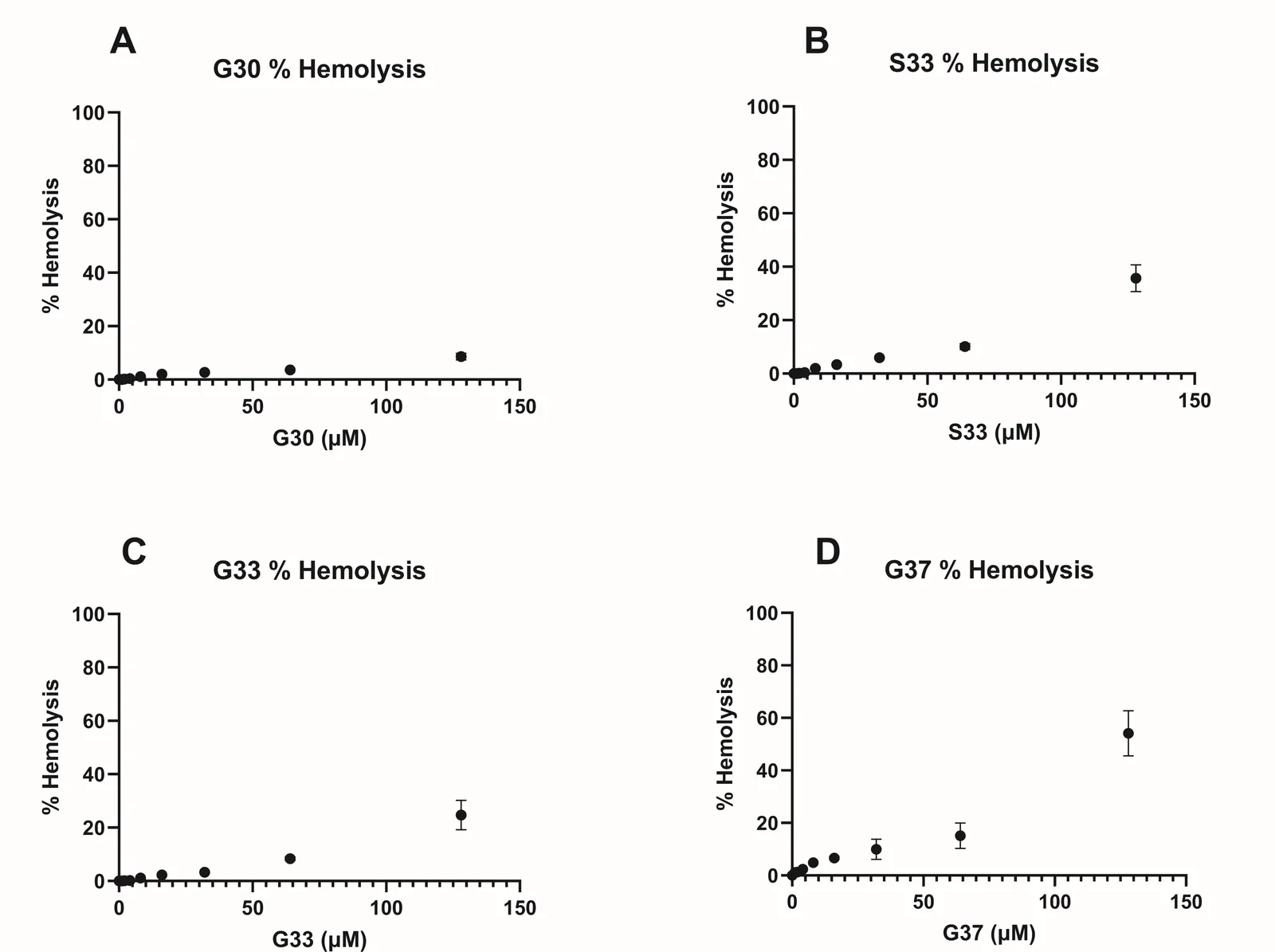
The percent hemolysis of sheep red blood cells from exposure to G30, S33, G33, and G37. Error bars represent the standard error of the mean. The estimated LD_50_ for (**A**) G30, (**B**) S33, and (**C**) G33 was not determined (>128 µM). (**D**) G37: LD_50_ was 121.25 µM. All experiments were performed in triplicate with three technical replicates. All experiments had a negative control (PBS) standardized to 0% lysis and a positive control (Triton X-100) standardized to 100% lysis (not shown).

The hemolytic activity of each peptide and reference was determined against defibrinated sheep red blood cells (Fig. 2). Peptide G30 displayed minimal hemolytic activity (Fig. 2A). G30, S33, and G33 did not reach 50% lysis at the highest concentration tested (128 µM), so their LD₅₀ values exceeded the assay range (>128 µM); among these, S33 (Fig. 2B) was slightly more toxic than G33 (Fig. 2C) at 128 µM. Only G37 reached a definable LD₅₀ (121.25 µM; Fig. 2D) and even so maintained low toxicity at the concentrations required for bacterial inhibition (<32 µM).

### Time-killing activity

**Fig 3:**
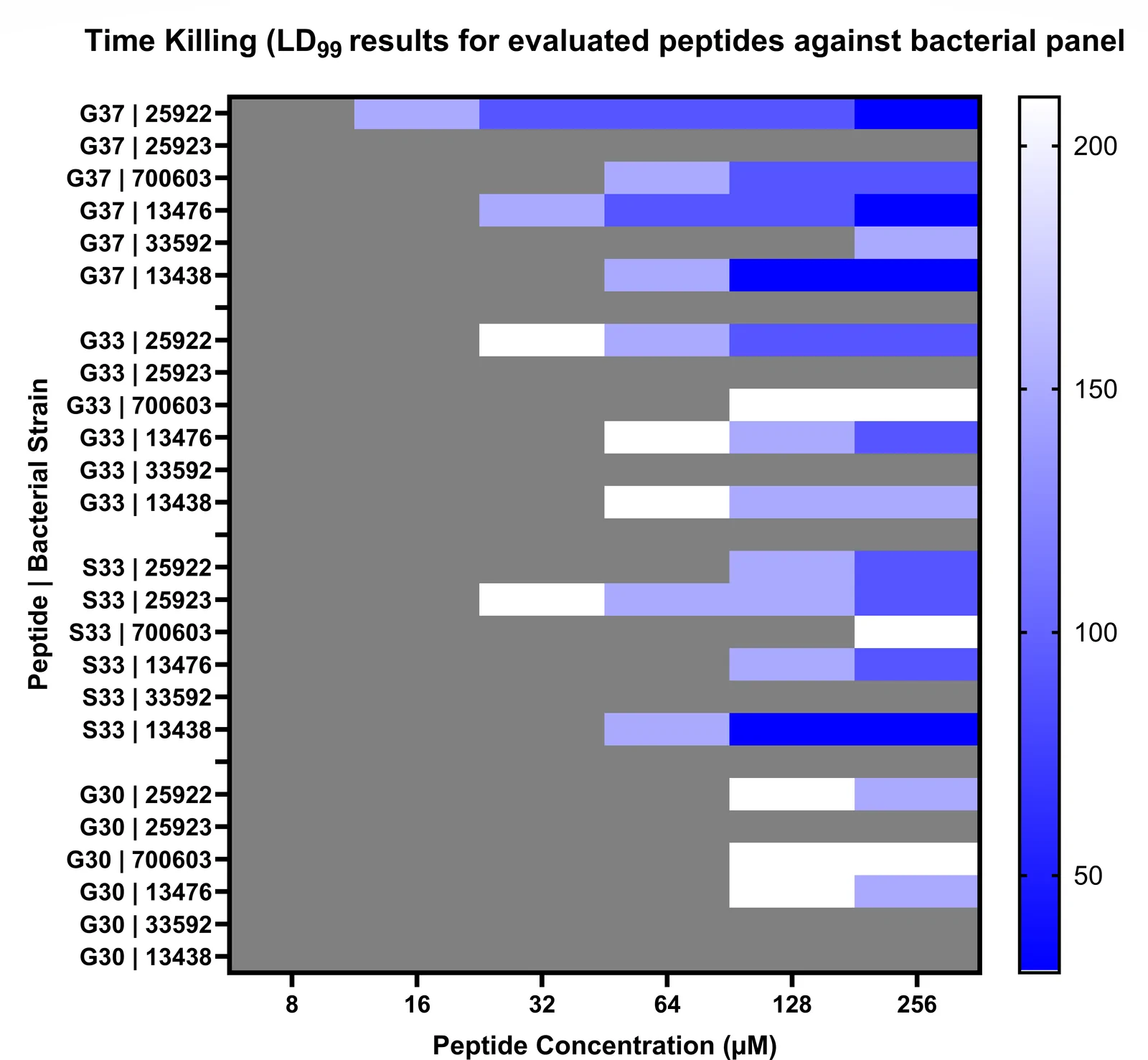
Heatmap of lethal dose (LD₉₉; <1 colony averaged) time points for G37, G33, S33, and G30 at peptide concentrations ranging from 8 to 256 µM against six bacterial strains. Each cell represents the time (min) required to achieve LD₉₉ at a given peptide concentration, determined in triplicate with three technical replicates. Dark blue indicates rapid killing (30 min); lighter shades indicate slower killing (up to 210 min). Gray cells indicate no killing (lawn growth) at the tested concentration and time points. G37 demonstrated the broadest and most rapid bactericidal activity, achieving LD₉₉ against five of six strains, including MRSA strain ATCC 33592 (150 min at 256 µM). G33 and S33 showed intermediate activity concentrated at higher peptide concentrations, while G30 displayed the narrowest-activity spectrum, with killing limited to three strains at ≥128 µM.

**Fig 4:**
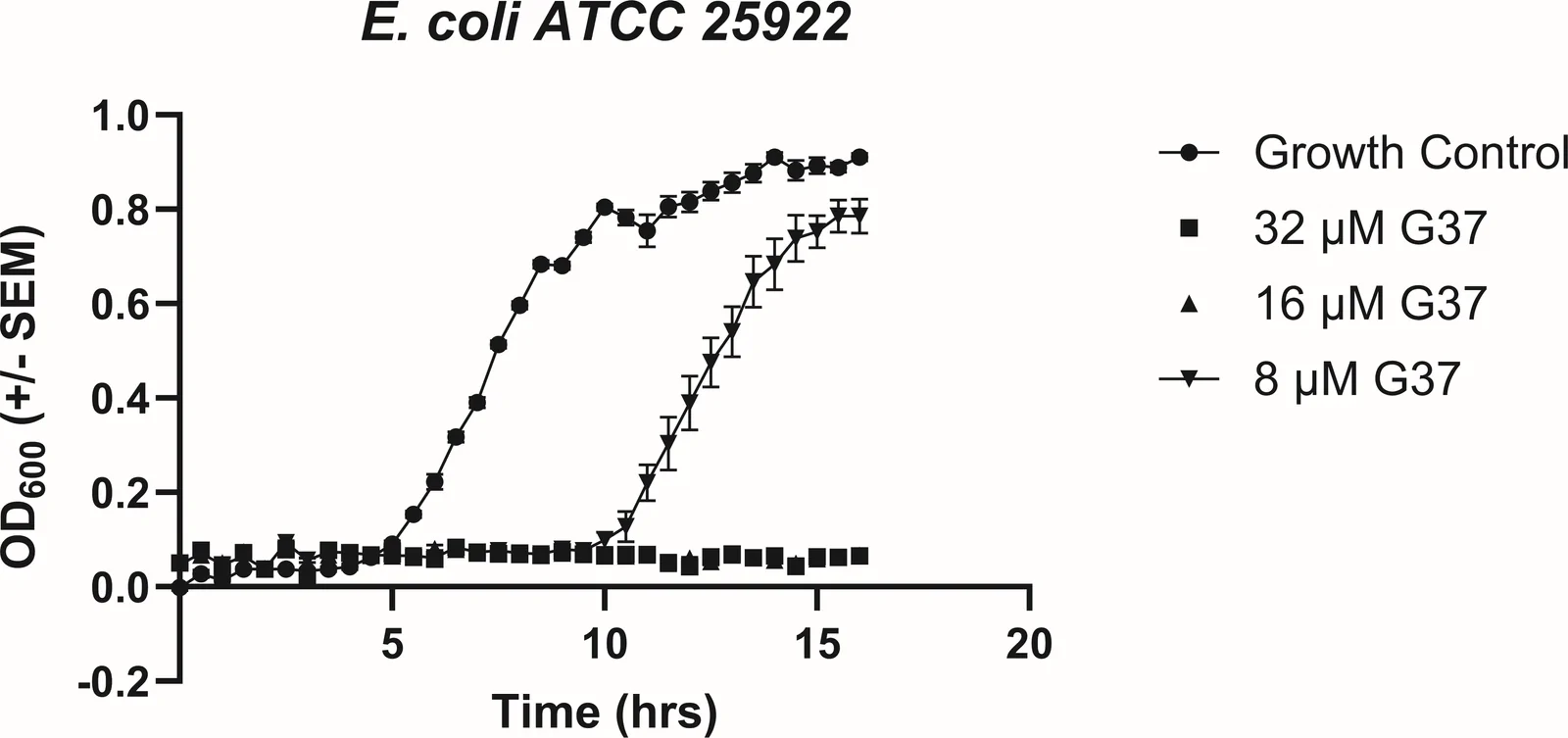
Growth curve for G37 against *E. coli* ATCC 25922 at 2× MIC (squares), 1× MIC (upward triangles), and 0.5× MIC (downward triangles). Growth curves were taken in triplicate with three technical replicates. The standard error of the mean informed the error bars. The growth control of Mueller-Hinton broth (circles) was compared against the three evaluated concentrations.

The time-killing activity of each peptide and reference was determined against reference strains of antibiotic-susceptible and -resistant bacteria (Table 2) to compare the efficiency of the peptides compared to traditional antibiotics. Peptide G30 displayed minimal inhibition of bacterial growth relative to the bacterial negative control, consistent with the MIC findings. However, G37 began to kill susceptible strains within 30 min and achieved full killing at 1× MIC within 150 min for all strains against which it was effective (Fig. 3). This is supported by the rapid growth shift observed at sub-MIC concentrations of G37 (Fig. 4). The rate of killing was slower for both G33 and S33 (slowed growth at 90 min and killed at 150/210 min; Fig. 3).

## DISCUSSION

With the emergence and continued spread of multidrug-resistant (MDR) microorganisms, the continued development and optimization of the therapeutics are critical. By taking advantage of compounds that have already been sculpted by natural selection, it may be possible to discover promising new candidates among the vast sequence space of antimicrobial peptides. Moreover, identifying physicochemical parameters that guide peptide activity against MDR organisms and influence off-target toxicity might inform the design of subsequent sequences for optimization purposes. This study showed that an *in silico* consensus-sequence-driven approach to the Brevinin-2 family could produce effective drug candidates, including against MDR bacteria. For example, our findings show that G37 inhibited the growth of both a carbapenem-resistant *E. coli* (NCTC 13476) at 16 µM and a KPC-3 carbapenemase-producing *K. pneumoniae* (NCTC 13438) at 64 µM. Although the heightened toxicity of some peptides remains a limiting factor (G37 = LD_50_ of 121.25 µM), this was not universally observed (G30, S33, and G33 = LD_50_ >128 µM, the highest concentration tested). Furthermore, while many traditional antibiotics take hours or days to kill a sufficient population of bacteria (34), G37 manifested rapid killing within 150 min, consistent with bactericidal activity.

Moreover, a standardized in-house systematic procedure of stepwise synthetic production allows for a potential economic advantage depending on the availability of materials and expertise to what is often a time-consuming and expensive task (35). The current study found this approach to provide peptide yields at >90% purity regardless of the length.

Further studies might address the potential differences in Brevinin-2 peptide selectivity for gram-negative and -positive bacteria, as suggested in S33 and G33. One reason for this selective preference is likely to be the sequence differences at the N-terminus, whereas the last ten residues were held constant between the two, including the Rana-box domain. This variation could allow the two sequences to bind with different affinity to different gram-positive and -negative bacterial membranes. However, the limited number of strains evaluated prevents broad claims about selective activity. Consequently, future work could perturb the Brevinin-2 sequences by replacing the N-terminus of G37 with the N-terminus of S33 to potentially target gram-positive organisms such as *S. aureus* to further validate selectivity trends against a broader strain panel.

Emerging evidence of combinatorial applications of AMPs with other antimicrobial agents demonstrates the synergistic potential of these compounds when combined with conventional antibiotics, allowing a reduction in the concentration of both compounds and thereby reducing host toxicity and inhibiting or evading bacterial resistance mechanisms (36–38). For example, when peptide MSI-78 was combined with colistin, a >4-fold decrease in the MIC was observed (39). Indeed, while potentiating traditional antibiotics is perhaps the most direct synergy with AMPs, secondary effects such as increased membrane permeability, biofilm disruption, and expanded spectrum range are of considerable importance (36). Although this was not evaluated here, it represents a potential avenue for future discovery.

Despite these positive attributes and the encouraging *in vitro* results displayed by G37, it is challenging to predict how the peptide would react *in vivo* due to environmental and physiological factors. For example, the 1-h exposure time of the hemolytic assay may underestimate longer exposure times that would occur *in vivo*. Several reports indicate that while some desired outcomes might be manifested with *in vivo* systems, other qualities may not meet expectations or may increase toxicity (40, 41). When considering the time-consuming and expensive nature of peptide synthesis, purification, evaluation, and validation, novel peptides are a daunting task facing the microbiological community. The nature of the work requires access and utilization of expensive, specialized equipment like peptide synthesizers and fast liquid chromatography instruments that are difficult to scale on the industrial level. This problem is exacerbated by the vast target space that needs to be addressed. For example, this work did not address the activity of the peptides against *Clostridioides difficile* or *Candida albicans*, two organisms of extreme concern due to their propensity for nosocomial infections (42, 43). Lastly, this work is strongly limited by the number of experimental combinations deemed attainable by the group performing the work. Even ten peptides, when tested against ten bacteria under five different conditions, necessitate five hundred unique experimental combinations. Despite these challenges, our approach begins to address a holistic approach toward exploring this alternative antibiotic space.

A notable limitation of this study is the absence of biofilm efficacy data. Clinically, biofilm-associated infections are of particular concern because bacteria within biofilm matrices can tolerate antimicrobial concentrations 10- to 1,000-fold higher than their planktonic counterparts, and the MIC values reported here reflect only planktonic susceptibility (44). Given that the therapeutic concentrations of G37 (16–64 µM) already approach the range at which moderate hemolytic activity was observed, the concentrations necessary for biofilm disruption or eradication may exceed acceptable toxicity thresholds. However, members of the Brevinin-2 superfamily have demonstrated the capacity to target biofilm-forming species in their planktonic state (11), suggesting that the membrane-disruption mechanism characteristic of these peptides may partially offset the concentration barrier imposed by biofilm architecture. Whether this property is retained in the consensus-derived peptides described here remains to be determined and represents a priority for future investigation. Such studies should include minimum biofilm eradication concentration (MBEC) assays alongside hemolytic profiling to establish whether a viable therapeutic window exists for biofilm-associated applications.

Although antibiotic resistance is a daunting challenge, antimicrobial peptides are a natural reservoir of potential therapeutics. However, to discover potential drugs in a timely fashion and produce them on a commercial scale, a standard approach to sequence design and peptide production is needed. Although significant challenges, such as toxicity and cost, remain for many peptide families and for the field, this study sought to exemplify a streamlined pipeline based on the Brevinin-2 family. Work remains to elucidate the mechanism of action of the Brevinin-2 family and how the local environment influences peptide-bacteria interactions. There is increasing evidence that the activity of AMPs is closely linked to membrane selectivity; hence, considering the membrane target during the design process is critical (45). The paradigm of Brevinin-2 peptide design, and potentially other AMPs, is the smooth transition from *in silico* to *in vitro* to *in vivo* for true success.

## SUPPLEMENTAL MATERIAL

Supplemental material aem.01553-26-s0001.pdf Fig. S1 to S9; Tables S1 to S4.

ASM does not own the copyrights to Supplemental Material that may be linked to, or accessed through, an article. The authors have granted ASM a non-exclusive, world-wide license to publish the Supplemental Material files. Please contact the corresponding author directly for reuse.
