# Supplementary material for: In vitro evaluation of novel consensus-sequence-generated Brevinin-2 antimicrobial peptides
Source: Appl Environ Microbiol. 2026 Sep 14;92(10):e01553-26. doi: 10.1128/aem.01553-26 (PMC13689303; doi:10.1128/aem.01553-26)
Supplement: Supplementary file 1 [file aem.01553-26-s0001.pdf]

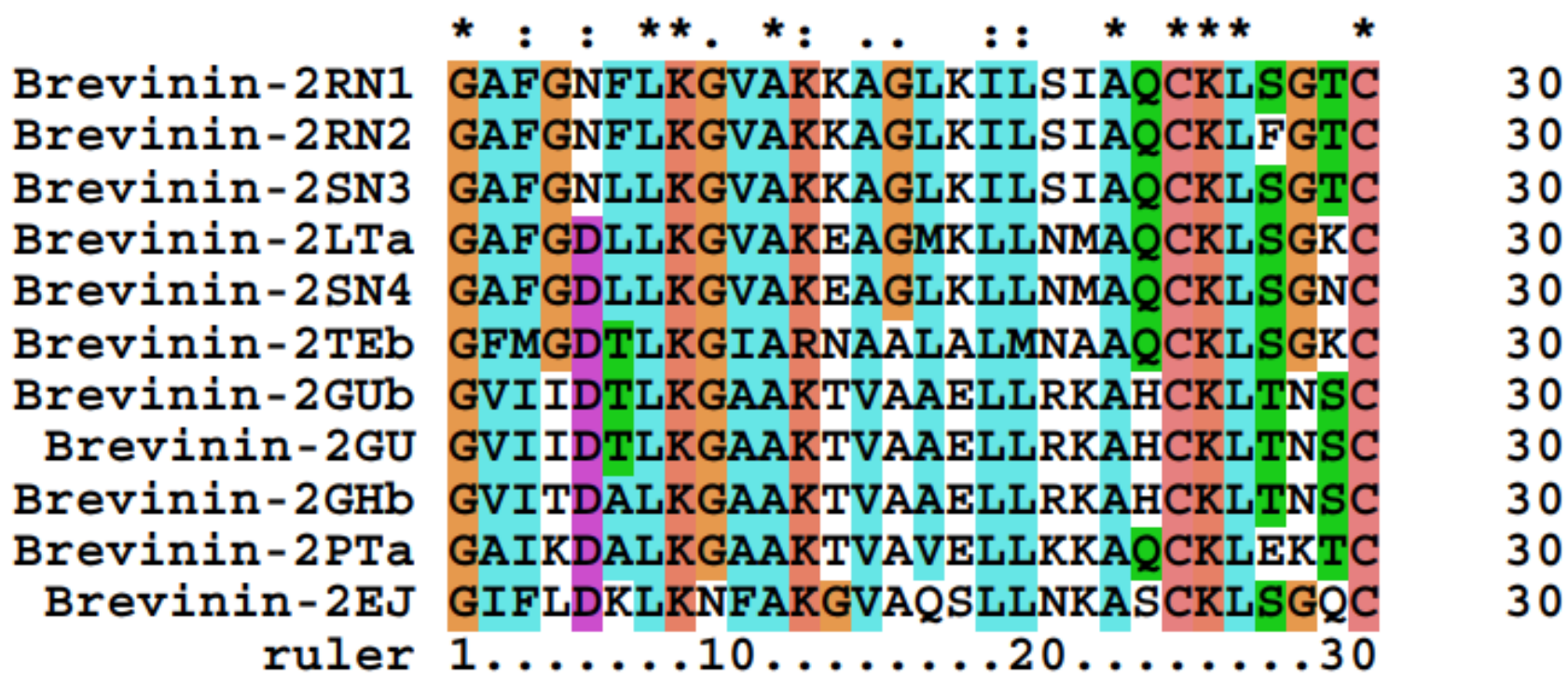

Figure S1: Multiple sequence alignment of 11 Brevinin-2 peptides (Group 1) with experimental antimicrobial activity using the ClustalX2 software obtained from the Antimicrobial Peptide Database and other public databases (12-17). Conserved amino acids are denoted with an asterisk (*). Colored amino acids denote their physicochemical identity; blue residues are hydrophobic, polar residues are green, negatively charged residues are purple, and positively charged residues are red. Critical residues such as cysteine, glycine, and aromatic residues are colored pink, orange, and cyan respectively. The length of each peptide is denoted with the rightmost column value. (https://www.jalview.org/help/html/colourSchemes/clustal.html)

```
                 *.:  ..*..  :  .  :.*.  .:*     **:  .  *
  Brevinin-2TP2 SILSTLKDVGISALKNAGSGVLKTLLCKLNKNCEK     35
  Brevinin-2TP1 SILSTLKDVGISAIKSAGSGVLSTLLCKLNKNC--     33
  Brevinin-2LF2 SIMSTLKQFGISAIKGAAQNVLGVLSCKIAKTC--     33
  Brevinin-2OR8 SFLDTLKNLAISAAKGAGQSVLSTLSCKLSKTC--     33
  Brevinin-2OR5 SFLDTLKNLAISAAKGAGQSVLSTLSCKLSETC--     33
  Brevinin-2OR9 SFLSTFKELAINAAKNAGQSILHTLSCKLDKTC--     33
 Brevinin-2OR10 SFLSTFKELAINAAKNAGQSLLHTLSCKLDKTC--     33
  Brevinin-2RE1 SFLSTFKKLAIDAAKNAGQSVLHTLTCKLDNQC--     33
  Brevinin-2ISb SFLTTFKDLAIKAAKSAGQSVLSTLSCKLSNTC--     33
  Brevinin-2ISa SLLDTFKNLAVNAAKSAGVSVLNALSCKISRTC--     33
  Brevinin-2OR1 SVLGTVKDLLIGAGKSAAQSVLTALSCKLSNSC--     33
  Brevinin-2OR4 SVLGTVKDLLIGAGKSAAQSVLTANSCKLSNSC--     33
  Brevinin-2ISc SVLGTVKDLLIGAGKSAAQSVLTTLSCKLSNSC--     33
 Brevinin-2HS2B SLLGTVKDLLIGAGKSAAQSVLKGLSCKLSKDC--     33
 Brevinin-2HS2A SLLGTVKDLLIGAGKSAAQSVLKGLSCKLSKDC--     33
  Brevinin-2OW2 SVMGTVKDLLIGAGKSAAQSVLKSLSCKLSNDC--     33
  Brevinin-2OW1 SVMGTVKDLLIGAGKSAAQSVLKSLSCKISNDC--     33
  Brevinin-2OW3 SVMGTVKDLLIGAGKSAAQSVLKALSCKLSKDC--     33
  Brevinin-2MS1 SFLDKFKDVAIGVAKGAGTGVLKALLCKLDNSC--     33
  Brevinin-2LTb SILDKIKNVALGVARGAGTGILKALLCKLDKSC--     33
          ruler 1.......10........20........30.....
```

Figure S2: Multiple sequence alignment of 20 Brevinin-2 peptides (Group 2) with experimental antimicrobial activity using the ClustalX2 software obtained from the Antimicrobial Peptide Database and other public databases (12-17). Conserved amino acids are denoted with an asterisk (*). Colored amino acids denote their physicochemical identity; blue residues are hydrophobic, polar residues are green, negatively charged residues are purple, and positively charged residues are red. Critical residues such as cysteine, glycine, and aromatic residues are colored pink, orange, and cyan respectively. The length of each peptide is denoted with the rightmost column value. (https://www.jalview.org/help/html/colourSchemes/clustal.html)

```
                 *.                    .   *:    *
Brevinin-2SKb    GLFNVFKKVGKNVLKNVAGSLMDNLKCKVSGEC-     33
Brevinin-2SKa    GLFSAFKKVGKNVLKNVAGSLMDNLKCKVSGEC-     33
  Brevinin-2T    GLLSGLKKVGKHVAKNVAVSLMDSLKCKISGDC-     33
 Brevinin-2Ob    GIFNVFKGALKTAGKHVAGSLLNQLKCKVSGEC-     33
 Brevinin-2Oa    GLFNVFKGALKTAGKHVAGSLLNQLKCKVSGGC-     33
Brevinin-2TSa    GIMSLFKGVLKTAGKHVAGSLVDQLKCKITGGC-     33
Brevinin-2DYd    GIFDVVKGVLKGVGKNVAGSLLEQLKCKLSGGC-     33
Brevinin-2DYc    GLFDVVKGVLKGVGKNVAGSLLEQLKCKLSGGC-     33
Brevinin-2DYb    GLFDVVKGVLKGAGKNVAGSLLEQLKCKLSGGC-     33
Brevinin-2LF1    GFMDTAKNVAKNVAKNVAVTLLDKLRCKVTGGC-     33
Brevinin-2OA4    GLLDTFKNLALNAAESAGVSVLNSLSCKLSKTC-     33
Brevinin-2GRa    GLLDTFKNLALNAAKSAGVSVLNSLSCKLSKTC-     33
Brevinin-2OR7    GLLDTIKNMALNAAKSAGVSVLNTLSCKLSKTC-     33
Brevinin-2OR3    GLIDTIKNMALNAAKSAGVSVLNTLSCKLSKTC-     33
Brevinin-2OR6    GLLDTIKNMALNAAKSAGVSVLNSLSCKDSKTC-     33
Brevinin-2OR2    GLLDTIKNMALNAAKSAGVSVLNSLSCKLSKTC-     33
Brevinin-2OA1    GILDTFKNMALNAAKSAGVSVLNALSCKLSKTC-     33
Brevinin-2OA2    GLLDTFKNMALNAAKSAGVSVLNALSCKLSKTC-     33
Brevinin-2OA3    GLLDTFKNMAINAAHGAGVSVLNALSCKLKKTC-     33
Brevinin-2OA5    GLLDGILNANFNAAKSAGTSVLNALSCKLSKTC-     33
Brevinin-2HSa    GLLDSLKNLAINAAKGAGQSVLNTLSCKLSKTC-     33
Brevinin-2GRb    GVLGTVKNLLIGAGKSAAQSVLKTLSCKLSNDC-     33
Brevinin-2OA7    GVLGTVKDLLIGAGKSAAQSTLKTLSCKISNDC-     33
Brevinin-2OA8    GVLATVKNLLNGTGDGAAQSVLKTLSCKLSNDC-     33
Brevinin-2OA6    GVLATVKNLLIGTGDGAAQSVLKTLSCKLSNDC-     33
 Brevinin-2Ta    GILDTLKNLAKTAGKGILKSLVNTASCKLSGQC-     33
  Brevinin-2E    GIMDTLKNLAKTAGKGALQSLLNKASCKLSGQC-     33
 Brevinin-2Ei    GILSTIKDFAIKAGKGAAKGLLEMASCKLSGQC-     33
Brevinin-2RTb    GILDTLKEFGKTAAKGIAQSLLSTASCKLAKTC-     33
Brevinin-2RTa    GLMSTLKDFGKTAAKEIAQSLLSTASCKLAKTC-     33
Brevinin-2CG1    GILDKLKEFGISAARGVAQSLLNTASCKLAKTC-     33
   Brevinin-2    GLLDSLKGFAATAGKGVLQSLLSTASCKLAKTC-     33
Brevinin-2SN2    GVLDTLKNVAIGVAKGAGTGVLKALLCQLDKSC-     33
Brevinin-2LTc    GVLDTFKDVAIGVAKGAGTGVLKALLCKLDKSC-     33
Brevinin-2SN1    GLRDKIKNVAIDVAKGAGTGVLKALLCQLDKSC-     33
Brevinin-2PTe    GFLDSFKNAMIGVAKSVGKTALSTLACKIDKSC-     33
Brevinin-2PTc    GLLDSFKNAMIGIAKSAGKTALNKIACKIDKTC-     33
Brevinin-2PTb    GFKGAFKNVMFGIAKSAGKSALNALACKIDKSC-     33
Brevinin-2GHk    GFSSLFKAGAKYLLKQVGKAGAQQLACKAANNC-     33
Brevinin-2GHa    GFSSLFKAGAKYLLKSVGKAGAQQLACKAANNCA     34
        ruler    1.......10........20........30....
```

Figure S3: Multiple sequence alignment of 40 Brevinin-2 peptides (Group 3) with experimental antimicrobial activity using the ClustalX2 software obtained from the Antimicrobial Peptide Database and other public databases (12-17). Conserved amino acids are denoted with an asterisk (*). Colored amino acids denote their physicochemical identity; blue residues are hydrophobic, polar residues are green, negatively charged residues are purple, and positively charged residues are red. Critical residues such as cysteine, glycine, and aromatic residues are colored pink, orange, and cyan respectively.

(https://www.jalview.org/help/html/colourSchemes/clustal.html)

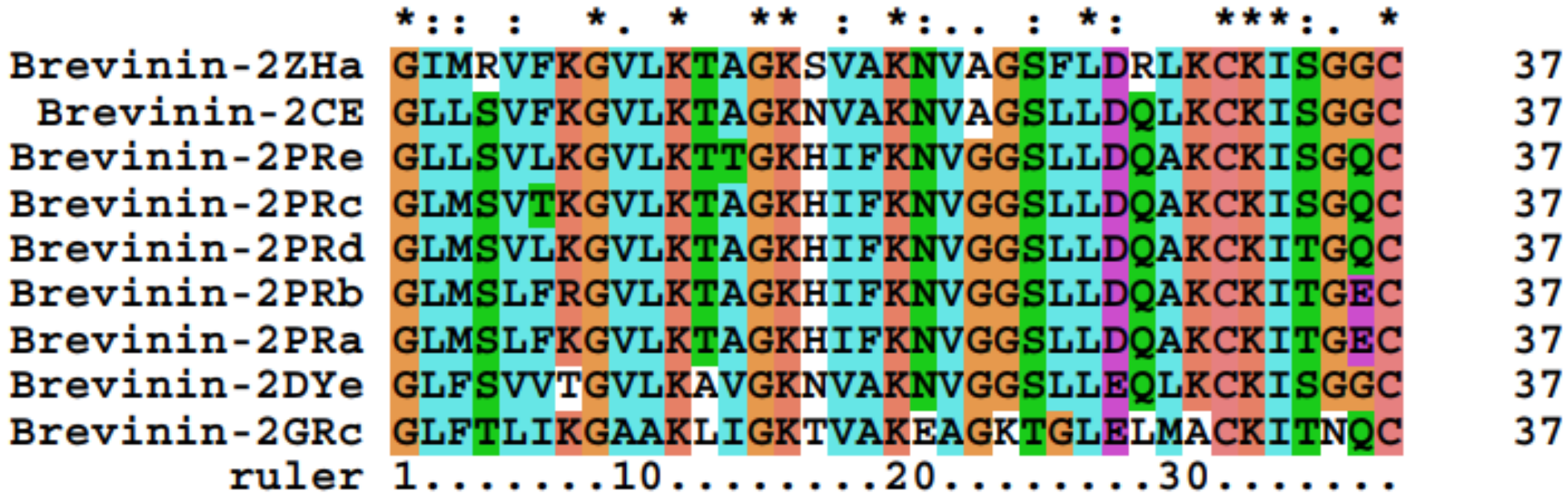

Figure S4: Multiple sequence alignment of 9 Brevinin-2 peptides (Group 4) with experimental antimicrobial activity using the ClustalX2 software obtained from the Antimicrobial Peptide Database and other public databases (12-17). Conserved amino acids are denoted with an asterisk (*). Colored amino acids denote their physicochemical identity; blue residues are hydrophobic, polar residues are green, negatively charged residues are purple, and positively charged residues are red. Critical residues such as cysteine, glycine, and aromatic residues are colored pink, orange, and cyan respectively. The length of each peptide is denoted with the rightmost column value. (https://www.jalview.org/help/html/colourSchemes/clustal.html)

	Residue Positional Frequency of Novel Brevinin-2 Peptide																													
	1	2	3	4	5	6	7	8	9	10	11	12	13	14	15	16	17	18	19	20	21	22	23	24	25	26	27	28	29	30
	G^{11}	A^{6}	F^{6}	G^{6}	D^{8}	T^{3}	L^{11}	K^{11}	G^{10}	V^{5}	A^{11}	K^{10}	T^{4}	A^{6}	A^{6}	L^{5}	K^{5}	L^{8}	L^{10}	N^{4}	K^{5}	A^{11}	Q^{7}	C^{11}	K^{11}	L^{11}	S^{6}	G^{7}	T^{4}	C^{11}
		V^{3}	I^{4}	I^{2}	N^{3}	L^{3}			N^{1}	A^{4}		R^{1}	K^{3}	V^{5}	G^{5}	A^{3}	E^{4}	I^{3}	M^{1}	S^{3}	I^{3}		H^{3}				T^{3}	N^{3}	S^{3}	
		F^{1}	M^{1}	T^{1}		F^{2}				F^{1}			E^{2}			M^{1}	A^{1}			R^{3}	M^{2}		S^{1}				F^{1}	K^{1}	K^{2}	
		I^{1}		L^{1}		A^{2}				I^{1}			N^{1}			V^{1}	S^{1}			K^{1}	A^{1}						E^{1}		Q^{1}	
				K^{1}		K^{1}							G^{1}			Q^{1}													N^{1}	
**Final Sequence**	**G**	**A**	**F**	**G**	**D**	**T**	**L**	**K**	**G**	**V**	**A**	**K**	**T**	**A**	**A**	**L**	**K**	**L**	**L**	**N**	**K**	**A**	**Q**	**C**	**K**	**L**	**S**	**G**	**T**	**C**

Table S1: Design of novel Brevinin-2 peptide designated G30 using a consensus-sequence approach. The positional frequency of each amino acid from conserved sequences of 11 peptides (Fig. S1) determines the placement of individual residues for the novel sequence.

	Residue Positional Frequency of Novel Brevinin-2 Peptide																																
	1	2	3	4	5	6	7	8	9	10	11	12	13	14	15	16	17	18	19	20	21	22	23	24	25	26	27	28	29	30	31	32	33
	S^{20}	F^{7}	L^{16}	G^{8}	T^{18}	V^{8}	K^{20}	D^{12}	L^{15}	A^{9}	I^{18}	G^{10}	A^{18}	A^{9}	K^{19}	S^{11}	A^{20}	G^{11}	Q^{15}	S^{15}	V^{17}	L^{20}	K^{8}	T^{9}	L^{19}	S^{15}	C^{20}	K^{20}	L^{17}	S^{12}	K^{10}	T^{7}	C^{20}
		V^{6}	M^{4}	S^{6}	K^{2}	F^{6}		N^{4}	V^{4}	L^{8}	V^{1}	S^{5}	V^{2}	G^{8}	R^{1}	G^{5}		A^{9}	S^{2}	G^{4}	I^{2}		S^{4}	A^{6}	N^{1}	L^{4}			I^{3}	D^{5}	N^{8}	S^{5}	
		I^{4}		D^{5}		L^{5}		E^{2}	F^{1}	G^{3}	L^{1}	N^{3}		I^{2}		N^{4}			T^{2}	N^{1}	L^{1}		T^{3}	S^{2}		T^{1}				N^{2}	E^{1}	D^{5}	
		L^{3}		T^{1}		I^{1}		Q^{1}				D^{1}		L^{1}					V^{1}				H^{3}	G^{2}						A^{1}	R^{1}	N^{2}	
								K^{1}				K^{1}											G^{1}	N^{1}								Q^{1}	
**Final Sequence**	**S**	**F**	**L**	**G**	**T**	**V**	**K**	**D**	**L**	**A**	**I**	**G**	**A**	**A**	**K**	**S**	**A**	**G**	**Q**	**S**	**V**	**L**	**K**	**T**	**L**	**S**	**C**	**K**	**L**	**S**	**K**	**T**	**C**

Table S2: Design of novel Brevinin-2 peptide designated S33 using a consensus-sequence approach. The positional frequency of each amino acid from conserved sequences of 20 peptides (Fig. S2) determines the placement of individual residues for the novel sequence.

	Residue Positional Frequency of Novel Brevinin-2 Peptide																															
	1	2	3	4	5	6	7	8	9	10	11	12	13	14	15	16	17	18	19	20	21	22	23	24	25	26	27	28	29	30	31	32	33
	G^{40}	L^{20}	L^{24}	D^{25}	T^{21}	F^{16}	K^{39}	N^{22}	V^{12}	A^{20}	K^{16}	N^{14}	A^{24}	A^{22}	K^{36}	S^{15}	A^{22}	G^{19}	V^{11}	S^{31}	L^{17}	L^{33}	N^{18}	T^{13}	L^{32}	S^{22}	C^{40}	K^{38}	L^{26}	S^{25}	K^{21}	T^{16}	C^{40}
		I^{9}	F^{7}	S^{7}	V^{6}	L^{9}	L^{1}	G^{7}	L^{9}	L^{10}	I^{12}	G^{12}	V^{10}	V^{15}	D^{2}	G^{13}	V^{15}	A^{18}	Q^{10}	G^{4}	V^{17}	M^{3}	K^{7}	Q^{8}	A^{7}	K^{9}		Q^{2}	I^{6}	D^{6}	G^{13}	G^{6}	
		V^{6}	M^{4}	N^{3}	S^{4}	V^{7}		D^{4}	M^{7}	G^{6}	L^{8}	T^{8}	I^{2}	I^{3}	H^{1}	N^{7}	I^{3}	L^{3}	G^{8}	T^{3}	A^{3}	V^{2}	D^{5}	A^{8}	I^{1}	A^{5}			V^{5}	A^{6}	N^{6}	D^{5}	
		F^{5}	S^{2}	G^{3}	L^{3}	I^{7}		K^{3}	A^{5}	M^{3}	F^{2}	Y^{2}	L^{2}		E^{1}	H^{3}			K^{7}	A^{2}	G^{2}	A^{2}	S^{4}	S^{5}		L^{3}			A^{2}	T^{2}		S^{5}	
			I^{1}	A^{2}	G^{2}	A^{1}		E^{2}	F^{5}	N^{1}	N^{1}	S^{1}	T^{2}			Q^{1}			T^{4}		T^{1}		E^{4}	K^{3}		R^{1}			D^{1}	K^{1}		E^{3}	
			R^{1}		A^{2}			A^{2}	G^{2}		A^{1}	H^{1}				E^{1}							Q^{2}	N^{2}								Q^{3}	
			K^{1}		K^{2}							D^{1}												M^{1}								N^{2}	
**Final Sequence**	**G**	**L**	**L**	**D**	**T**	**F**	**K**	**N**	**V**	**A**	**K**	**N**	**A**	**A**	**K**	**S**	**A**	**G**	**V**	**S**	**L**	**L**	**N**	**T**	**L**	**S**	**C**	**K**	**L**	**S**	**K**	**T**	**C**

Table S3: Design of novel Brevinin-2 peptide designated G33 using a consensus-sequence approach. The positional frequency of each amino acid from conserved sequences of 40 peptides (Fig. S3) determines the placement of individual residues for the novel sequence.

	Residue Positional Frequency of Novel Brevinin-2 Peptide																																			
	1	2	3	4	5	6	7	8	9	10	11	12	13	14	15	16	17	18	19	20	21	22	23	24	25	26	27	28	29	30	31	32	33	34	35	36	37
	G^{9}	L^{8}	M^{5}	S^{7}	V^{6}	F^{4}	K^{7}	G^{9}	V^{8}	L^{8}	K^{9}	T^{7}	A^{6}	G^{9}	K^{9}	H^{5}	I^{5}	F^{5}	K^{9}	N^{8}	V^{8}	G^{7}	G^{8}	S^{8}	L^{7}	L^{9}	D^{7}	Q^{7}	A^{5}	K^{8}	C^{9}	K^{9}	I^{9}	S^{5}	G^{8}	Q^{4}	C^{9}
		I^{1}	L^{2}	T^{1}	L^{3}	L^{2}	T^{1}		A^{1}	A^{1}		A^{1}	T^{1}			N^{2}	V^{4}	A^{4}		E^{1}	A^{1}	A^{2}	K^{1}	T^{1}	F^{1}		E^{2}	D^{1}	L^{3}	A^{1}				T^{4}	N^{1}	G^{3}	
			F^{2}	R^{1}		T^{1}	R^{1}					L^{1}	V^{1}			T^{1}									G^{1}			E^{1}	M^{1}							E^{2}	
						V^{1}							I^{1}			S^{1}																					
						I^{1}																															
**Final Sequence**	**G**	**L**	**M**	**S**	**V**	**F**	**K**	**G**	**V**	**L**	**K**	**T**	**A**	**G**	**K**	**H**	**I**	**F**	**K**	**N**	**V**	**G**	**G**	**S**	**L**	**L**	**D**	**Q**	**A**	**K**	**C**	**K**	**I**	**S**	**G**	**Q**	**C**

Table S4: Design of novel Brevinin-2 peptide designated G37 using a consensus-sequence approach. The positional frequency of each amino acid from conserved sequences of 9 peptides (Fig. S4) determines the placement of individual residues for the novel sequence.

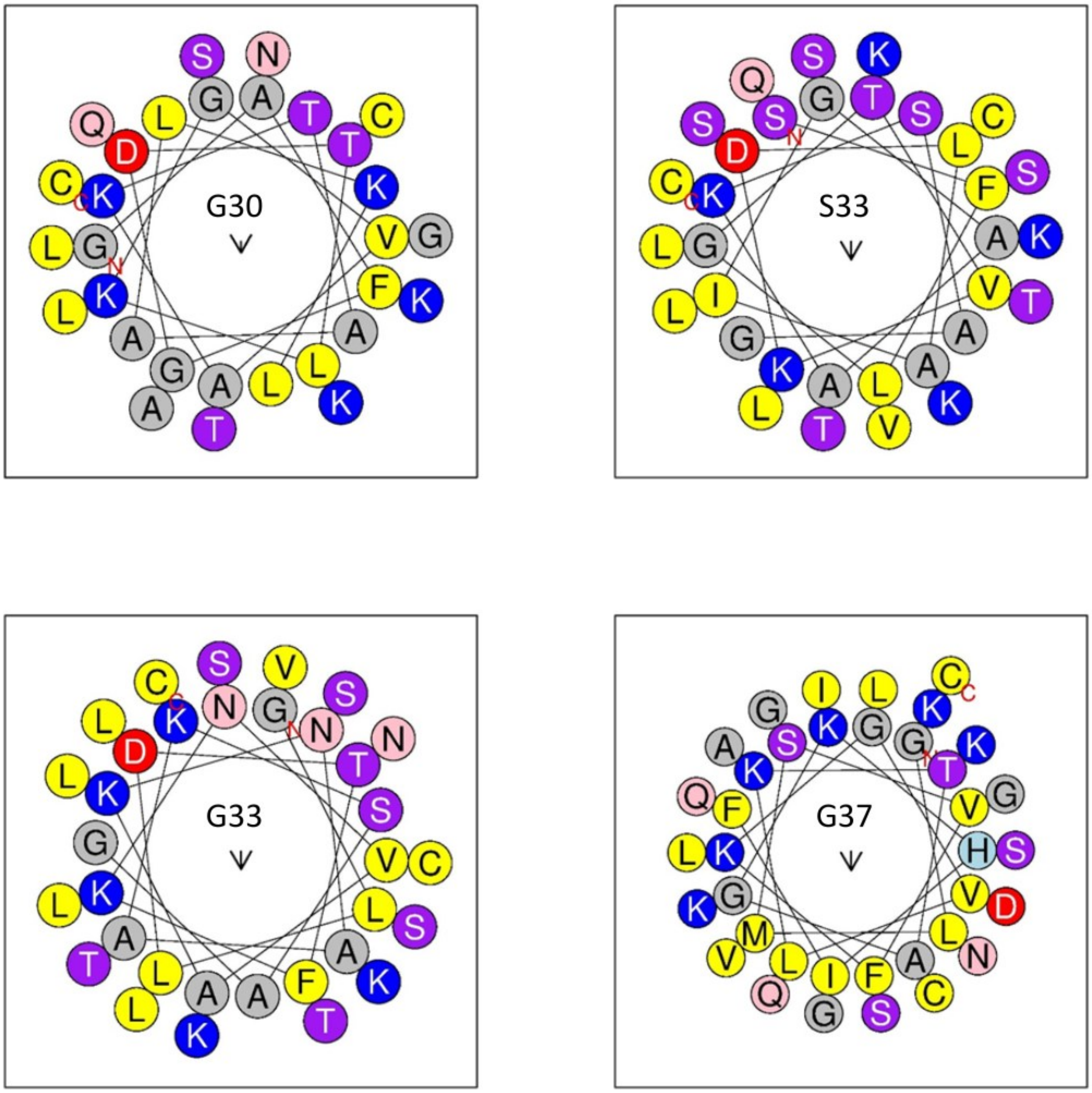

Figure S5: Helical wheel diagrams of Brevinin-2 G30, S33, G33, and G37 using Heliquest software. All negatively-charged, positively-charged, hydrophilic, and hydrophobic residues are colored by red, blue, purple, and yellow respectively.

(https://heliquest.ipmc.cnrs.fr/index.html)

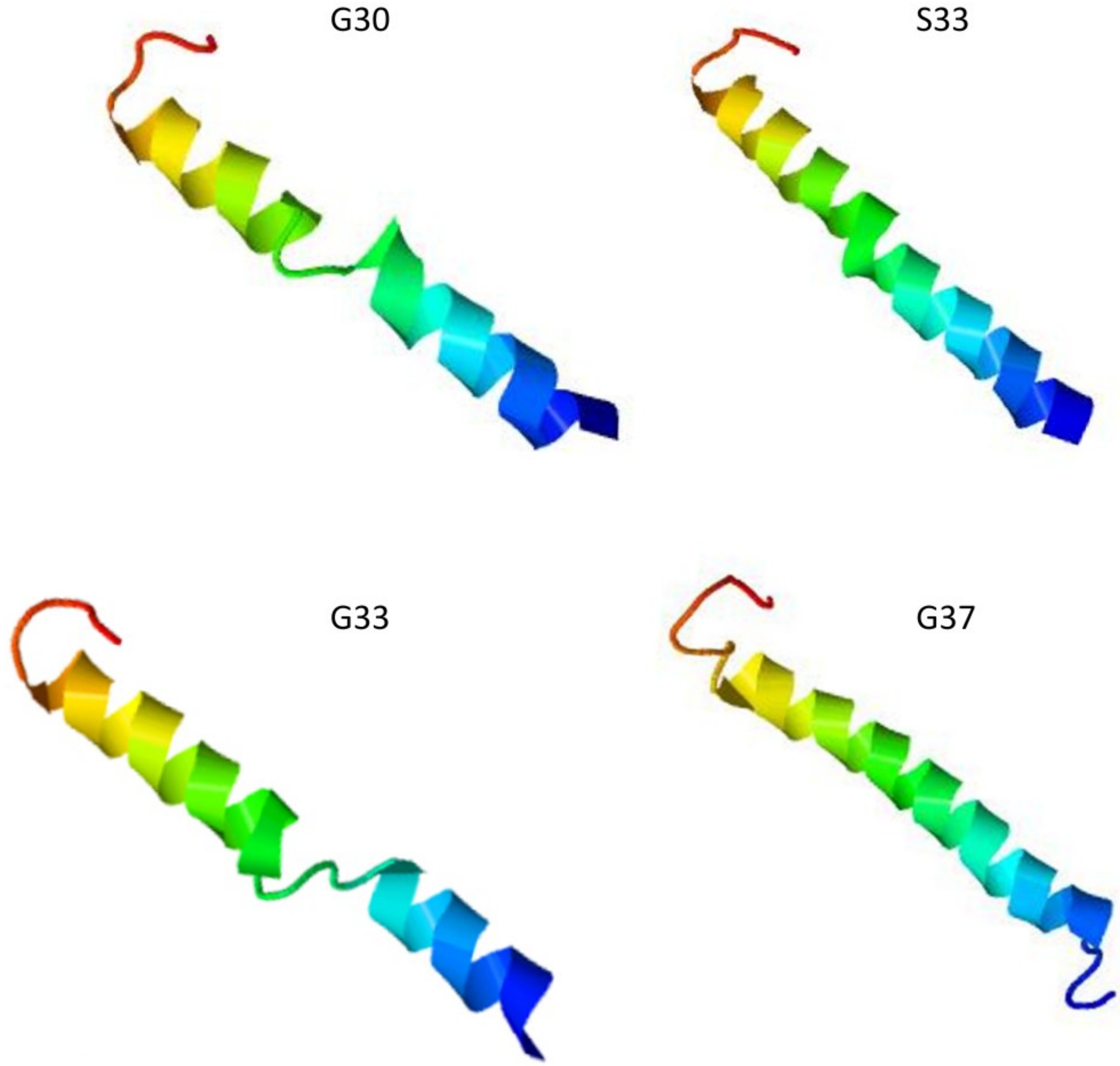

Figure S6: Predicted three-dimensional structures of G30, S33, G33, and G37 using ribbon diagrams from I-TASSER software. Color moves from the blue N-terminus to the orange C-terminus. (https://zhanggroup.org/I-TASSER/)

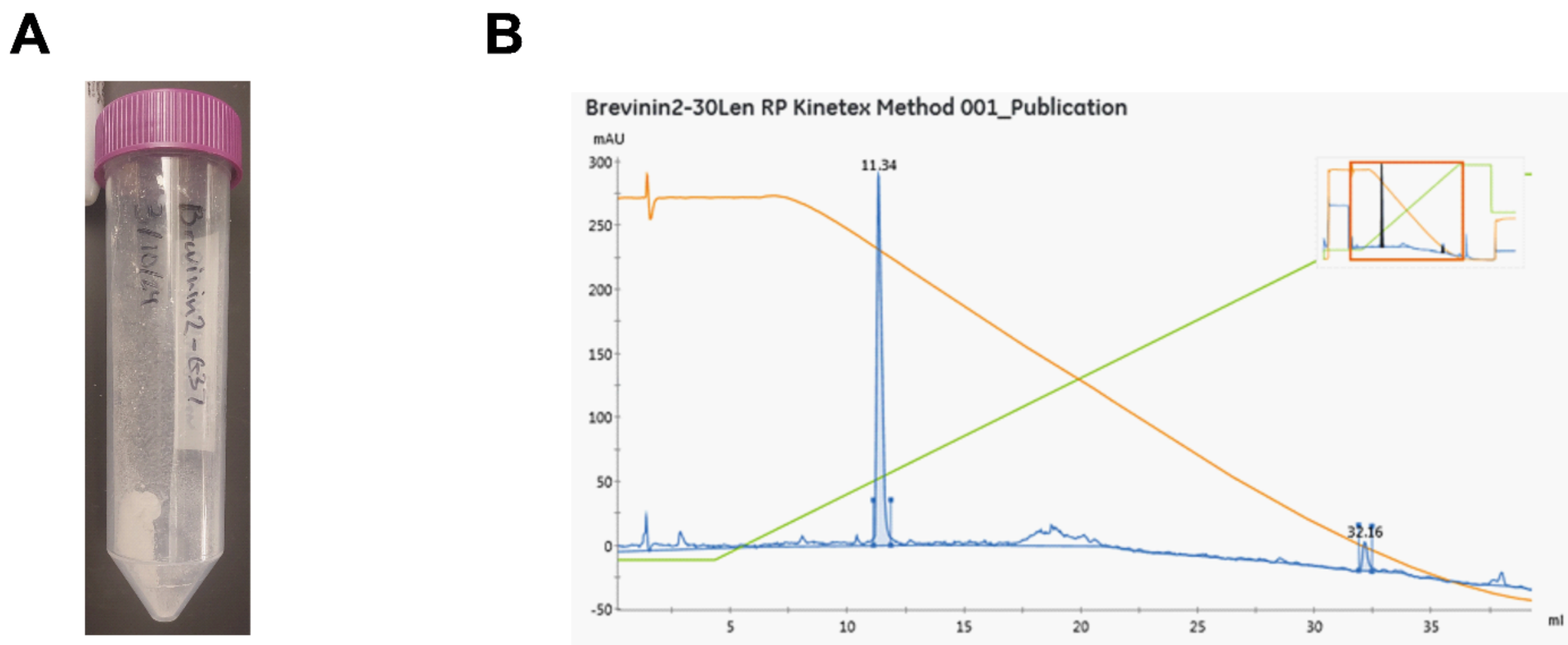

Figure S7. Representative image of (A) peptide powder following synthesis, cleavage, and purification alongside the representative confirmation (B) analytical chromatogram. The chromatogram shown is magnified on the column loading and elution stages of purification. All peptides were synthesized, cleaved, and purified using the same methods. All chromatograms were taken using a standard RP-FPLC approach.

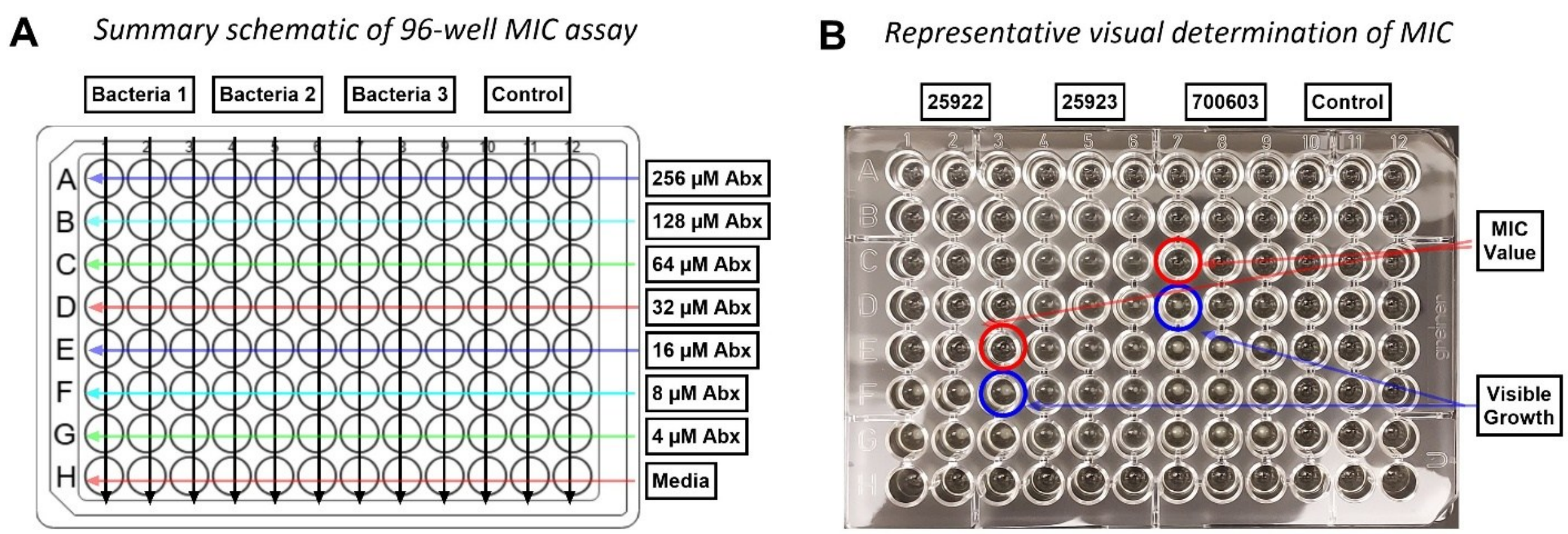

Figure S8. Representative (A) schematic and (B) visual determination of minimum inhibitory concentrations for all tested peptides and antibiotics.

A

Representative time killing activity of G37 against four targets

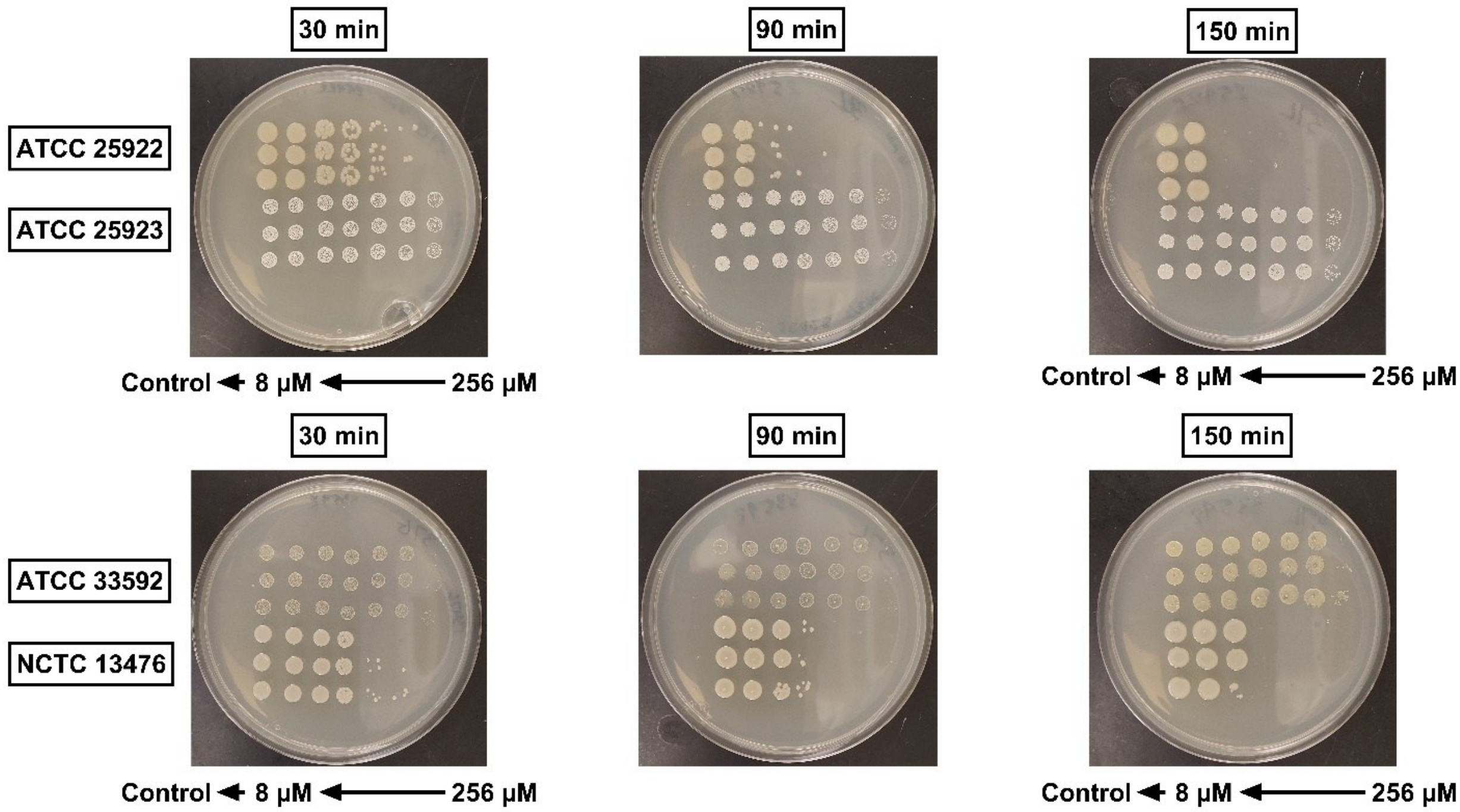

B

Time-Killing (LD_{99}) results for evaluated peptides against bacterial panel

Conc. G37 (µM)	LD_{99} (min)					
	25922	25923	700603	13476	33592	13438
0	Lawn	Lawn	Lawn	Lawn	Lawn	Lawn
8	Lawn	Lawn	Lawn	Lawn	Lawn	Lawn
16	150	Lawn	Lawn	Lawn	Lawn	Lawn
32	90	Lawn	Lawn	150	Lawn	Lawn
64	90	Lawn	150	90	Lawn	150
128	90	Lawn	90	90	Lawn	30
256	30	Lawn	90	30	150	30

Conc. G30 (µM)	LD_{99} (min)					
	25922	25923	700603	13476	33592	13438
0	Lawn	Lawn	Lawn	Lawn	Lawn	Lawn
8	Lawn	Lawn	Lawn	Lawn	Lawn	Lawn
16	Lawn	Lawn	Lawn	Lawn	Lawn	Lawn
32	Lawn	Lawn	Lawn	Lawn	Lawn	Lawn
64	Lawn	Lawn	Lawn	Lawn	Lawn	Lawn
128	210	Lawn	210	210	Lawn	Lawn
256	150	Lawn	210	150	Lawn	Lawn

Conc. G33 (µM)	LD_{99} (min)					
	25922	25923	700603	13476	33592	13438
0	Lawn	Lawn	Lawn	Lawn	Lawn	Lawn
8	Lawn	Lawn	Lawn	Lawn	Lawn	Lawn
16	Lawn	Lawn	Lawn	Lawn	Lawn	Lawn
32	210	Lawn	Lawn	Lawn	Lawn	Lawn
64	150	Lawn	Lawn	210	Lawn	210
128	90	Lawn	210	150	Lawn	150
256	90	Lawn	210	90	Lawn	150

Conc. S33 (µM)	LD_{99} (min)					
	25922	25923	700603	13476	33592	13438
0	Lawn	Lawn	Lawn	Lawn	Lawn	Lawn
8	Lawn	Lawn	Lawn	Lawn	Lawn	Lawn
16	Lawn	Lawn	Lawn	Lawn	Lawn	Lawn
32	Lawn	210	Lawn	Lawn	Lawn	Lawn
64	Lawn	150	Lawn	Lawn	Lawn	150
128	150	150	Lawn	150	Lawn	30
256	90	90	210	90	Lawn	30

Conc. GUb (µM)	LD_{99} (min)					
	25922	25923	700603	13476	33592	13438
0	Lawn	Lawn	Lawn	Lawn	Lawn	Lawn
8	Lawn	Lawn	Lawn	Lawn	Lawn	Lawn
16	Lawn	Lawn	Lawn	Lawn	Lawn	Lawn
32	210	Lawn	Lawn	210	Lawn	Lawn
64	150	150	Lawn	210	Lawn	Lawn
128	90	150	210	150	Lawn	150
256	90	90	150	90	Lawn	90

Blank Control	LD_{99} (min)					
	25922	25923	700603	13476	33592	13438
0	30	30	30	30	30	30
8	30	30	30	30	30	30
16	30	30	30	30	30	30
32	30	30	30	30	30	30
64	30	30	30	30	30	30
128	30	30	30	30	30	30
256	30	30	30	30	30	30

Bacteria Control	LD_{99} (min)					
	25922	25923	700603	13476	33592	13438
0	Lawn	Lawn	Lawn	Lawn	Lawn	Lawn
8	Lawn	Lawn	Lawn	Lawn	Lawn	Lawn
16	Lawn	Lawn	Lawn	Lawn	Lawn	Lawn
32	Lawn	Lawn	Lawn	Lawn	Lawn	Lawn
64	Lawn	Lawn	Lawn	Lawn	Lawn	Lawn
128	Lawn	Lawn	Lawn	Lawn	Lawn	Lawn
256	Lawn	Lawn	Lawn	Lawn	Lawn	Lawn

Figure S9. The time-killing kinetics of G37, G33, S33, and G30 under decreasing peptide concentrations against the standard bacterial panel. (**A**) After exposure to a peptide or control for a set amount of time, a visual quantification was taken of the lethal dose (LD_{99}; <1 colony averaged) on Mueller-Hinton plates. (**B**) The time necessary to obtain a lethal dose against a specific organism at a given peptide concentration was taken in triplicate with three technical replicates, with any growth higher than a lethal dose labelled as 'Lawn.' Included are the Brevinin-2GUb control, blank (no bacteria) control, and bacteria (no treatment) control. This was used to create the heatmap of **Figure 3**.
